# Retraction Note: Ethaselen synergizes with oxaliplatin in tumor growth inhibition by inducing ROS production and inhibiting TrxR1 activity in gastric cancer

**DOI:** 10.1186/s13046-022-02268-7

**Published:** 2022-02-15

**Authors:** Haiyong Zhang, Jing Wu, Jinqiu Yuan, Huafu Li, Yawei Zhang, Wang Wu, Wei Chen, Chunming Wang, Sijun Meng, Songyao Chen, Mingyu Huo, Yulong He, Changhua Zhang

**Affiliations:** 1 Digestive Diseases Center, The Seventh Affiliated Hospital, Sun Yat-sen University, Shenzhen, 518107 Guangdong China; 2 Department of Gastrointestinal Surgery, The First Affiliated Hospital, Sun Yat-sen University, Guangzhou, 510080 Guangdong China; 3 Clinical Research Center, The Seventh Affiliated Hospital, Sun Yat-sen University, Shenzhen, 518107 Guangdong China; 4 Department of Pathology, The Seventh Affiliated Hospital, Sun Yat-Sen University, Shenzhen, 518107 Guangdong China

**Retraction Note: J Exp Clin Cancer Res 40, 260 (2021)**

**https://doi.org/10.1186/s13046-021-02052-z**

This article was published in error and has been removed.

All authors agree to this retraction.

